# Mental Health, Alcohol and Substance Use of Refugee Youth

**DOI:** 10.3389/fpsyt.2021.713152

**Published:** 2021-11-19

**Authors:** Jelena Vasic, Roberto Grujicic, Oliver Toskovic, Milica Pejovic Milovancevic

**Affiliations:** ^1^Clinic for Children and Adolescents, Institute of Mental Health, Belgrade, Serbia; ^2^Laboratory for Experimental Psychology, Department of Psychology, Faculty of Philosophy, University of Belgrade, Belgrade, Serbia; ^3^Faculty of Medicine, University of Belgrade, Belgrade, Serbia

**Keywords:** alcohol and substance use, refugee youth, adolescents, PTSD, abuse and neglect, trauma

## Abstract

This study aims to explore the prevalence of alcohol and substance use among young refugees along with the indicators of experienced psychological difficulties. It is based on a sample of 184 children and adolescents aged 11–18 years old, residing at two refugee centers in the Republic of Serbia. Out of 184 participants, the majority was male (*N* = 155; 84.29%). More than a half of participants (53.3%) displayed significant symptoms of PTSD. 50% consume energy drinks, 28% use tobacco; 13% use alcohol; 4.6% use marijuana; 1.7% use LSD, amphetamines, glue, tranquilizers and cocaine. Female respondents were more frequently expressing emotional difficulties (*p* < 0.05) while male participants were more frequent users of alcohol or substances (*p* < 0.01). Younger children were more frequently expressing symptoms of hyperactivity and prosocial behavior, while they were less frequently using substances. There is also a significant negative correlation between the years of education and individual proneness to substance use. Furthermore, those who resided in a greater number of refugee camps were found to experience greater levels of emotional and behavioral difficulties and face a greater risk of physical abuse. The burden of migration increases proneness to substance use, as a consequence of scarce coping resources and the stress of adjusting. Migrants are vulnerable to substance use, since some of them have commonly witnessed and/or personally experienced pre-and post-migration stress and trauma, including loss of homes and livelihoods, violence, torture and family separation. Preventive programs need to focus on the problem of alcohol and substance use among this vulnerable population.

## Introduction

A growing number of children and youth belong to the refugee population. They either migrate unaccompanied across national and international borders or are separated from their families during transition. According to the UNHCR report, global resettlement needs increased to a record of 1.4 million refugees in 2021 (1). UNICEF estimates that 10,000 unaccompanied and separated refugee children are in need of care globally (2). Between January and August 2020, nearly 50,000 refugees and migrants (25% children) arrived in Europe (1). In May 2021, the total number of refugees and migrants in the Republic of Serbia was estimated to be 5,603, of whom 4,550 were placed in reception or asylum centers, and 890 of them were not registered in any centers. Data available from the Serbian Commissariat for Refugees and Migration (SCRM) shows that there were 367 children in reception or asylum centers in Serbia in May of 2021; 247 boys and 120 girls, while 67 of these were unaccompanied and separated children (UASC). UASC are refugee children who have been separated from both parents and other relatives and are not being cared for by any adult who is, by law or custom, responsible for doing so (3).

Refugee children and youth should be considered as highly vulnerable to the development of behavioral abnormalities, functional deficits, and psychiatric disorders since they are arriving in host countries with an alarming “risk burden.” Some of the most important social-based stress factors for this population include financial hardships, exposure to violence, discrimination and stigmatization, linguistic barriers, limited access to health-care, as well as a lack of cultural sensitivity of service providers (4, 5). In addition, UASC are frequently victims of forced labor, drug trafficking, human trafficking and sexual exploitation (6).

Family- and community-level risk factors for mental health problems among resettled refugee children and youth include being unaccompanied and separated, or living in a single-parent household, having parents with psychiatric problems, exposure to family violence, economic hardship, perceived discrimination in the country of origin, as well as post-migration violence (7, 8). A mental health survey conducted on the population of refugees in Serbia in 2017 has shown that 88.5% of the asylum seekers placed in centers for asylum reported having mental health problems, whereas 2/3 displayed high psychological vulnerability (9).

Post-traumatic stress disorder (PTSD) is often discussed in relation to refugees' mental health. Studies conducted on refugee children aged 8 years old or younger show that up to 80% of participants experience depression, anxiety, and PTSD, as well as behavioral challenges (10). Shedding light on the interaction between trauma, PTSD and the development of substance use disorders in the refugee population is of particular interest to researchers. Researchers of PTSD and substance disorder comorbidity suggest that drug use and abuse might be the result of efforts to medicate the symptoms. Nonetheless, the possibility for shared vulnerability could not be ruled out (11).

The risk of substance use among refugee children and youth is being discussed from several viewpoints. Relevant literature emphasizes the difficulty in generalizing results given that exposure to risk factors depends on the nature of their flight, the way they were welcomed by their host country, and the degree of cultural similarity to the host society (12). The risk of abuse and exploitation among refugee children and youth is significantly higher in UASC (5). Stealing, drug smuggling and sex work were specified as the main sources of income, as there is a lack of access to regular jobs, due to refugee status and age boundaries (13). War experience and separation from family are recognized as the main sources of distress in the domain of mental health. In relation to the social support system, the main stressors of these children include the lack of engagement with peers, unawareness of the skills they can use to protect themselves, fear of kidnapping and sexual abuse (14).

The primary aim of this study is to explore various socio-demographic and psychological indicators of mental health problems among the young refugee population in Serbia, along with determining the prevalence of alcohol and substance abuse in this population.

## Methods

### Sample

This research is based on a sample of 184 children and adolescents aged 11–18 years old, placed in asylum centers in Serbia. Out of 184 participants, the majority were male (*N* = 155; 84.29%), while 29 participants (15.71%) were female. Besides gender, the sample is also divided into age groups—the first group of children aged from 11 to 14 years (48.02%, *N* = 90) and one group aged from 15 to 18 years (51.08%, *N* = 94). In total, only 40 participants (21.7%) were accompanied by their parents and 12% (*N* = 22) by the members of their close family, while others traveled with other familiar and/or unfamiliar people.

### Procedure

#### Data Collection

Data collection was carried out in two asylum centers in Serbia, in the period from October 2020 to February 2021. The first center (Krnjaca) was primarily the center for families with children, while the second center (Bogovadja) was the center for UASC aged 11–18 years. In Krnjaca, the interviewers were approaching potential participants (children aged 11–18) and their parents, asking if they would like to participate in the research. In Bogovadja, interviewers recruited participants strictly in the presence of their custodians. The process of sampling was partly conducted using the snowball method, since the participants whose parents/custodians agreed to participate were encouraged to invite friends or relatives of appropriate age to participate. Almost all the children who were approached took part in the research. Very few of those who started the questioning (<10) opted out before completion, mostly due to the inability to understand the translator or simply due to lacking motivation. The respondents were divided into small groups, where their privacy for filling out questionnaires was assured. The data were collected directly from children using self-report questionnaires. As the majority of participants were illiterate, the translators read out the questions to participants in their native languages (Arabic, Pashtu or Farsi) and assisted in noting the responses, writing/circling the participants' responses on paper. The whole process was supervised by research coordinators. The interviewers were medical trainees in general psychiatry or child psychiatry with previous experience in work with children and adolescents. Nonetheless, prior to the fieldwork, they were additionally trained by experts in child and adolescent mental health.

### Ethics

This study has been approved by the Ethics Committee of the Institute of Mental Health, and has been performed in accordance with the principles of good clinical practice (No1060/2089/1). All of the participants, with their parents or legal custodians had signed the consent form prior to taking part in the research. For UASC, their custodian of the local Centre for Social Work signed the informed consent and was present during the whole assessment.

### Instruments

#### General Information Questionnaire

The general information questionnaire was specifically designed for this study with the aim of covering most relevant socio-demographic information. The questions were constructed to obtain information on the age, gender, country of origin and education level of children/adolescents, as well as to establish whether they were enrolled in formal education in the Republic of Serbia. We also assessed their current asylum status and with whom did they travel. In addition, the questionnaire captured their thoughts about the living conditions in asylum centers, how they perceive their safety and the availability of support in camps.

#### Strengths and Difficulties Questionnaire-Youth Self-Report Measure (SDQ-YR1)

The Strengths and Difficulties Questionnaire (SDQ) is a brief emotional and behavioral screening questionnaire appropriate for children and young people up to 18 years of age. The 25 items in the SDQ comprise 5 scales of 5 items each. The scales include the: emotional symptoms subscale, conduct subscale, hyperactivity or inattention subscale, peer relationships problem subscale, and prosocial behavior subscale. Each subscale is comprised of five questions, related to symptoms perceived over the previous 6 months, answered with a three-point Likert-type scale. These scales capture the internalizing and externalizing difficulties in children and young people, which is a configuration appropriate for epidemiological research on at-risk populations. According to the previous research, the SDQ showed good concurrent and discrimant validity (15, 16).

#### Adolescent Alcohol and Drug Involvement Scale

The Adolescent Alcohol and Drug Involvement Scale (AADIS) was developed as a research and evaluation tool to measure the extent of drug involvement in adolescents. It is a commonly used standardized instrument for measuring substance and alcohol use of underage young individuals (from 11 to 18 years). The first part of the questionnaire (scale A) measures the frequency of substance and alcohol use or abuse, and the second part (scale B) is focused on the overall impact of use on multiple domains of the child's functioning. It can be administered individually or in smaller groups. Results of previous studies indicate acceptable internal consistency (alpha = 0.85). AADIS scores correlated highly (e.g., *r* = 0.72) with self-reported levels of drug use, with subjects' perceptions of the severity of their own drug use problem (*r* = 0.79), and with clinical assessments (*r* = 0.75) (17).

#### Short Child Maltreatment Questionnaire

The Short Child Maltreatment Questionnaire (SCMQ) was developed by the World Health Organization (18). It includes seven items that were adapted from validated measurements for the specific purpose of creating a short questionnaire. It can be administered to adolescents and young people aged 10–18. The SCMQ does not aim to measure every type of maltreatment. Items reflect four dimensions—physical, emotional, and sexual abuse, and neglect—and a fifth dimension of witnessing parental physical violence. Questions endeavor to distinguish between moderate and severe levels, and single and frequent occurrences, of physical and emotional abuse. They aim to differentiate between physical and emotional neglect and contact and penetrative sexual abuse, although psychometric testing and validation are required to determine performance in practice. The SCMQ focuses on child maltreatment measuring only acts of violence against children by those in a position of power or trust.

#### The Children's Impact of Event Scale CRIES-1

The Impact of Events Scale (IES) was originally developed by Horowitz et al. (19) to monitor phenomena of re-experiencing a traumatic event and avoiding such experiences, as well as the feelings it gives rise to. Thus, the present version is designed for use with children aged 8 years and above who are able to read independently. The questionnaire is designed to measure three domains of PTSD reactions with three sub-scales including: intrusion—difficulties in staying asleep and dissociative experiences (like flashbacks); avoidance—the tendency to avoid thoughts or reminders about the incident; and arousal—feelings of irritation, anger, and difficulty in falling asleep. Psychometric data relevant to the reliability and validity of the eight-item version were presented in Yule (20).

### Data Analysis

For descriptive data analyses frequencies and percentages, or means and SD were used. Differences between groups were calculated using *t* tests, ANOVA and χ^2^ tests (with the subsequent Sidak *post-hoc* testing when needed). A multiple regression analysis was performed to assess the potential prediction of alcohol and substance use on AADIS. In order to predict PTSD diagnosis based on other scales we used the binary logistic regression analysis.

## Results

### Sociodemographic Data

The socio-demographic data are presented in Table 1. The majority of our respondents (72.3%; *N* = 133) were accompanied by familiar people. However, a third of the participants (27.2%; *N* = 50) were accompanied by people who were complete strangers to them. The vast majority of our participants were from Afghanistan (79.9%; *N* = 147). They were mostly living in rural areas prior to migration (58.2%; *N* = 107).

**Table 1:** Socio-demographic data of the respondents.

**Socio-demographic characteristics**	**Variable**	***N***	**%**	**Mean**	**SD**	**Missing data (%)**
Gender	Male	155	84.29			/
	Female	29	15.71			
Age				14.06	2.28	/
Years of education				6.7	3.11	/
Time away from home (months)				16.89	191.043	1.8
School enrolment	Yes	16	8.7			12
	No	146	79.3			
Country of origin	Afghanistan	147	79.9			0.1
	Iran	7	3.8			
	Pakistan	3	1.6			
	Syria	14	7.6			
	Iraq	8	4.3			
	Other	5	2.7			
Accompanied by	Parents	40	21.7			0.5
	Family members	22	12.0			
	Relatives	6	3.3			
	Friends	55	29.9			
	Familiar people	10	5.4			
	Unfamiliar people	50	27.2			
Asylum status	Accepted	13	7.1			0.5
	Rejected	9	4.9			
	On hold	7	3.8			
	Doesn't exist	133	72.3			
	I don't know	21	11.4			

For many of the respondents (60.9%; *N* = 112) the current refugee asylum center where the research was conducted was also their first center of residence. For the rest of the 72 participants, 23.1% of them have previously resided in one center, 41.5% in two centers, 10.8% in three centers, 4.6% in four, while 20% were in five or more centers. Among the participants, only 8.7% (*N* = 16) were enrolled in schools or formal/non-formal education in Serbia, while 79.3% (*N* = 146) of participants were not enrolled in the school system at all. For the remaining participants (12%; *N* = 22) data on education is missing. According to the public data of the Commissariat for Refugees and Migration of the Republic of Serbia, all the children placed in the Krnjaca Asylum Centre were enrolled in primary education. However, because of the COVID-19 pandemic, schooling was mostly conducted online, which could affect the children's perception of whether they were enrolled in the official school system. Meanwhile, most of the children and adolescents who were placed in the Bogovadja Asylum Centre were not enrolled in school, as most of them are of high school age.

#### Emotional and Behavioral Problems of Young Refugees

Approximately half of the respondents (49.4%; *N* = 91) were showing significant difficulties in peer relations. On the emotional symptoms scale, 30.4% (*N* = 56) were showing difficulties, while conduct problems were significant in 29.3% (*N* = 54) of children. 19.6% of children had difficulties with hyperactivity and inattention (*N* = 36). On the Total difficulty scale, 37% (*N* = 68) showed significant difficulties in overall functioning. On the Prosocial scale, a cut-off in accordance with the 10th percentile was pursued, since low ratings of prosocial skills indicate difficulties in this area and as low ratings were observed for one fifth of the participants (21.2%; *N* = 39). The results are presented in Table 2.

**Table 2:** Emotional and behavioral problems in young refugee population.

**Variable**	**Cut-off**	**Min**	**Max**	**Mean**	**SD**
Emotional symptoms	5	0	10	4.21	2.42
Conduct problems	3	0	10	2.60	2.21
Hyperactivity/inattention	5	0	10	3.64	2.135
Peer problems	3	0	8	3.37	1.851
Prosocial scale	6	3	10	7.95	1.805
Total difficulties scale (without prosocial)	15	0	34	13.80	6.29

*Self-rated with the Strengths and Difficulties Questionnaire (SDQ)*.

Next, the analysis was performed on the SDQ total impact scale. Since there are no cut-off criteria for this supplemental material, a score of 1 or above was regarded as a cut-off. Mean response for the total impact scale was 3.1511 (SD = 2.668). There were two significant negative correlations with SDQ domains and the age of participants; the younger the participants the more hyperactivity/inattention difficulties they had (*r* = 0.203; *p* < 0.05), but also expressed more prosocial behavior (*r* = 0.17; *p* < 0.05). Additionally, Emotional, Hyperactivity and Total Difficulty scales correlated positively with the number of camps in which the participants were placed (*r* = 0.286; *r* = 0.294; 0.277, respectively; *p* < 0.05). Girls experienced significantly greater emotional difficulties in comparison to boys (*t* = −2.294; df = 173; *p* < 0.05). Emotional difficulties on the SDQ Emotional scale (*F* = 3.386; *p* < 0.05) depend on who accompanied the child. The group which traveled with unfamiliar people had significantly higher scores on the Emotional difficulties scale compared to the group traveling with friends and familiar people (mean diff = −1.132; *p* < 0.05).

#### Substance and Alcohol Use

According to our collected data, a total of 50% (*N* = 88) of respondents consumed energy drinks, 28% (*N* = 46) smoked tobacco, 13% (*N* = 24) consumed alcohol, 4.6% (*N* = 7) smoked marijuana, 2.7% (*N* = 5) tried LSD, 2.29% (*N* = 4) amphetamines, 1.7% (*N* = 3) powder cocaine, 1.7% (*N* = 3) inhalants (e.g., glue, gasoline), 1.7% (*N* = 3) tranquilizers and 1% (*N* = 2) rock cocaine.

When the respondents were asked how frequently they use substances on a Likert scale from 0 (never) to 7 (several times a day), a significant difference in consumption habits was found between boys and girls. Boys reported consuming alcohol and substances significantly more often than girls (*t* = 2.953; *p* < 0.05). The sample was then divided into three groups according to the participants' companions on the journey. The first group included participants traveling with unfamiliar people, the second with friends and familiar people, and the third with family members. The analysis (one-way ANOVA) showed that the participants differ significantly in their scores on AADIS A scale (*F* = 7.731; *p* < 0.001). The *post hoc* analysis shows the greatest differences between the participants who were traveling with members of their family and the participants who traveled with unfamiliar people, who scored significantly higher on the AADIS A scale (mean diff = −3.62; *p* < 0.001), which is demonstrated in Table 3.

**Table 3:** Significance of differences according to who accompanied the child on AADIS A scale.

**Accompanied by**	***N***	**Mean**	**SD**	**F**	**df1; df2**	***p***
Family	65	2.40	3.08	7.73	2; 174	0.001
Friends	64	3.91	4.56			
Unfamiliar	48	6.02	6.77			

Multiple regression analysis was applied to predict alcohol and substance use according to other performed variables. Significant predictors were emotional abuse (beta = 0.268; *t* = 2.779; *p* < 0.01) and total impact of behavioral and emotional difficulties on their life (beta = 0.219; *t* = −2.239; *p* < 0.05). More emotional abuse leads to more alcohol and substance use; in contrast higher impact on SDQ scale leads to less alcohol and substance use (R^2^ = 0.221; *F* = 2.336; *p* < 0.05), which is presented in Table 4.

**Table 4:** Emotional abuse and higher impact on SDQ scale as predictors of alcohol and substance use.

	**Beta**	***t***	***p***
SCMQ_emotional_abuse	0.27	2.78	0.007
SDQ_impact	−0.22	−2.24	0.027

#### Abuse and Neglect

Forty percent of respondents (40.2%; *N* = 59) reported physical abuse by their parents or other family members. 28.6% (*N* = 42) experienced abuse at some point in their life, and 11.6% (*N* = 17) were abused in the last 12 months. Experience of emotional abuse was reported by 18.2% (*N* = 27); 15.5% (*N* = 23) experienced it at some point in their life, while 2.7% (*N* = 4) in the 12 months prior to questioning. Sexual abuse was reported by 8.8% (*N* = 13). Of those admitting to being sexual abused, 1% (*N* = 2) respondents reported it occurring in the last 12 months. Every fourth respondent was physically neglected (25.6%; *N* = 38). Out of those 25%, 7.4% (*N* = 11) were neglected in the last 12 months. Almost half of the respondents felt that they were emotionally neglected by the adults in their life (45.8%; *N* = 66); 14.5% said that this was the case in the last year. Only 14.5% (*N* = 21) reported that they were witnesses of abuse in the family; and 5.5% (*N* = 8) experienced it in the last year.

The strongest correlation is between the number of reception/asylum centers in which the children have been previously and physical abuse (*r* = 0.448; *p* < 0.001). Emotional abuse correlated positively with the number of months spent outside the country of origin (*r* = 0.301; *p* < 0.05). It is also established that children coming from rural areas experienced more emotional neglect (*t* = −1.976; df = 140.77; *p* < 0.05) and children of more educated parents experienced more emotional abuse (*t* = −2,572; df = 120; *p* < 0.05).

#### PTSD

Over a half of respondents had PTSD (53.3%; *N* = 98). Intrusion and Avoidance correlated positively with the total number of reception/asylum center in which the respondents resided (*r* = 0.330, *p* < 0.05; *r* = 0.279; *p* < 0.05). As it is shown in Table 5, participants that resided in two or more reception/asylum centers had significantly higher scores on all three CRIES scales: intrusion (*t* = −2.946; *p* < 0.001), avoidance (*t* = −3.237; *p* < 0.001) and arousal (*t* = −3.181; *p* < 0.001). Furthermore, participants who had PTSD also had significantly higher scores on several SCMQ scales—the physical abuse scale, the emotional neglect scale and the witness to parental abuse scale.

**Table 5:** Significance of number of reception/asylum center and PTSD development differences on CRIES and SCMQ scales.

		**N**	**Mean**	**SD**	**T**	**df**	**p**
CRIES_intrusion	One camp	110	7.41	4.76	−2.95	176	0.004
	Two or more camps	68	9.72	5.58			
CRIES_avoidance	One camp	110	8.08	5.31	−3.30	176	0.001
	Two or more camps	68	10.97	6.23			
CRIES_arousal	One camp	110	7.97	5.34	−3.18	176	0.002
	Two or more camps	68	10.84	6.57			
SCMQ_physical_abuse	No PTSD	61	1.39	0.94	−3.94	145	0.000
	With PTSD	86	2.17	1.33			
SCMQ_emotional_	No PTSD	59	1.63	1.14	−3.00	143	0.003
neglect	With PTSD	86	2.29	1.41			
SCMQ_parentabuse_	No PTSD	61	1.11	0.45	−2.65	143	0.009
witness	With PTSD	84	1.50	1.07			

A binary logistic regression analysis was used to predict a PTSD diagnosis according to the CRIES criteria, which is presented in Table 6. As a result, scores on witnessing parent abuse according to the SCMQ scale (B = 1.210; SE = 0.521; Wald = 5.390; *p* < 0.05) and the Total impact scale on the SDQ questionnaire (B = 0.277; SE = 0.097; Wald = 8.072; *p* < 0.05) significantly predict PTSD with accuracy of 71.4%. Children and adolescents witnessing family abuse have 3.3 times greater chances of developing PTSD, whereas children whose lives are more impacted by personal emotional and behavioral difficulties have 1.3 times greater chances of developing PTSD. Furthermore, participants who had PTSD also had significantly higher scores on several SCMQ scales—the physical abuse scale (*t* = −3.944; *p* < 0.001); the emotional neglect scale (*t* = −2.996; *p* < 0.01) and the witness to parental abuse scale (*t* = −2.645; *p* < 0.01).

**Table 6:** Significance of logistic coefficients for PTSD prediction.

	**Wald**	**df**	**Sig**.	**Exp(B)**
SCMQ_parentabuse_witnes	5.39	1	0.020	3.35
SDQ_impact	8.07	1	0.004	1.32

## Discussion

In the past decade, many research projects examined the occurrence of mental health problems in displaced populations. This study was conducted on a sample of young refugees representative of the underage refugee population currently residing in the Republic of Serbia. About one third of study participants were unaccompanied, and only 8.7% were enrolled in schools. In this research, we found that UASC were more prone to substance and alcohol use and reported more emotional difficulties compared to accompanied youths. According to the existing literature, the UASC more frequently show patterns of internalizing behavior as well as lower self-esteem in comparison with the children accompanied by parents (21). Younger UASC show more behavioral and emotional distress in comparison to unaccompanied adolescents (22). In a recent study also conducted in Serbia, similar findings were reported. The authors stated that UASC in Serbia most frequently reported that they suffered from anxiety, adjustment difficulties and PTSD, while accompanied youths surprisingly had slightly higher rates of depression and behavioral symptoms (23). However, it is important to emphasize that psychological distress of UASC depends on various factors that they are being exposed to during their transition and resettlement. During their transition, UASC usually move in groups and are sometimes supported by adults who are not their family members, and despite numerous challenges, they succeed in building flexibility and resilience (23). It is also noted in literature that psychological distress among UACS was lower when they were placed in high support living arrangements in their resettlement (24). On the other hand, accompanied refugee children and youth, even when they are in an intact family, are influenced by family dynamics and their parents' mental health (8, 23, 25). We can summarize that proximity of parents, relatives and familiar adults is a strong risk or protective factor for children's mental health.

The most commonly used substances among out participants were energy drinks, tobacco, alcohol, followed by other illegal substances such as marijuana, LSD, amphetamines, cocaine, inhalants (e.g., glue, gasoline) and tranquilizers. The most significant risk factors for alcohol and substance use include being male, unaccompanied, and separated from families, as well as a lack of education, and emotional abuse. It is noteworthy to compare these results with the results from the European School Survey Project on Alcohol and Other Drugs (ESPAD), that was conducted in 2015, and showed that the use of psychoactive substances in the population of 16-year-old students was present in 8.0% of the sample examined (26). Also, they found a higher incidence of substance use among boys than girls, which is concordant to our findings, and the most used illegal substance was cannabis (26). A significant number of our participants experienced PTSD symptoms as well as emotional and behavioral difficulties, but we found no statistically significant association between these kinds of difficulties and a propensity to alcohol and substance use. The assessment of alcohol and illegal drugs use in displaced populations is usually challenging due to many reasons (e.g., fear, cultural differences, etc.). Due to the same challenges, we also noted a low response rate on the scales that measured the frequency and severity of substance use. In our opinion, this was mainly due either to the perceived expectations of the Islamic religion, or fear of a breach of anonymity and that a truthful answer could possibly lead to legal issues. It is also possible that participants were hesitant to answer these questions, since illicit use of alcohol and substances can be associated with antisocial behavior or interpreted as a sign of weakness or vulnerability (27). In contrast, the research on substance use among the child and youth refugee population has grown significantly in recent years. Substance use itself is understood as an outcome of complex interactions between risk factors and protective factors. Some of the risk factors include being unaccompanied, coming from a specific alcohol/drug native culture, boredom and unemployment, traumatic experiences, poverty, and poor knowledge about treatment services (28). Protective factors, however, include being united with family, satisfying integration into the new society, good physical and mental condition, early identification of mental health needs and the availability of psychosocial services (28). Alcohol and substance use among young refugees is often discussed in relation to mental health problems and their potential bidirectional association: alcohol and substances could be used as self-medication, but also, they could have an additive effect on the etiopathogenesis of mental health problems. Findings of relevant literature suggest that untreated mental health problems, stressful living conditions, and a lack of support and control might put unaccompanied refugee children at risk of substance use (27). PTSD had previously been established as a strong lifetime indicator of an increased risk to alcohol use disorders and drug abuse/dependence (12, 29). However, in this research no statistically significant association between PTSD symptoms and a propensity to alcohol and substance use was found, possibly due to a low response rate.

Our findings also suggest that the consumption of alcohol and substances is significantly lower in young people who had been in two or more reception or asylum centers, compared with young people who were in their first reception or asylum center. Since Serbia is far away from their countries of origin, this could mean that they had spent most of the journey time on the road, and it had been difficult for them to access proper child protection services. At the same time, access to services could be delayed because it was challenging to reach them. We could also consider the possibility that children who had not been sheltered in reception or asylum center settings could be more exposed to harmful influences and experiences, especially if they were accompanied by strangers.

Our findings showed that duration of education contributes to the reduction of the proneness to substance abuse. The enrolment in school represents a critical source of protection, as it reduces exposure to early marriage, child labor, sexual exploitation, involvement in illicit activity, etc. Schools are also crucial for child protection as school staff can recognize children facing risk of abuse, or suspect that they are having mental health problems, and could help them to connect to appropriate services (30). The vast majority of examined subjects were not enrolled in the local education system either due to the COVID-19 pandemic or because of an anticipated short stay in Serbia (for many, Serbia is a transit country on the path to Western European countries).

Refugee children are also coping with the burden of many adverse life events, which could lead to the genesis of PTSD symptomatology. In our study, the children and adolescents who witnessed family abuse were more prone to developing PTSD. It is also established that a stronger impact of emotional and behavioral problems on children's life could be a predictive factor of PTSD. Almost half of the respondents have shown significant difficulties in peer relations, and about one third displayed significant difficulties in overall functioning. Bronstein and Montgomery's review of 22 studies on the mental health of refugee children and adolescents in high-income countries showed the prevalence of PTSD from 19 to 54% (average of 36%), and the prevalence of depression-specific symptoms from 3 to 30% (average of 18%). A few studies conducted on refugee children aged 8 years and less show that up to 80% experience depression, anxiety, and PTSD, as well as behavioral challenges (10). Further research is needed to understand the relationship of traumatic experience and alcohol and substance use, but evidence-based interventions that are pointed to coping mechanisms could also be helpful (31).

We also found high rates of all forms of abuse and neglect among our participants. This finding is in concordance with previous studies which report that the risk of abuse and exploitation among young refugees high, however significantly higher in unaccompanied children (5). This refers to abuse and violence that could have been experienced in their country of origin, on their journey and in the resettlement country. All forms of exploitation and abuse usually manifest in places where protective services are overstretched or non-existent. Some risk factors described include a shortage of living space, which forces children to cohabit with adults in crowded settings, and a general lack of safety and security in the facilities, including inadequate support by adults and services, and a lack of safe housing options (32).

This study had certain methodological limitations. The first limitation refers to the cross-sectional study design that does not allow conclusions on causal relationships. A history of substance abuse, PTSD symptoms or any other indicators cannot provide conclusive information on what are the causes and consequences or these indicators. Furthermore, a retrospective self-report as a method of data collection raises the challenge of recall bias, meaning that current memories of past experiences may not be as correct and objective. Also, the self-report method increases the chance for bias in terms of response set (random filling in, yes- and -no answers etc.). A significant challenge in conducting this research were language and cultural barriers. Finally, this study may be limited by selection bias: permission by parents or motivation by peers could affect one's likelihood to participate.

## Conclusion

Currently millions of children are displaced from their country of origin due to war persecution and poverty. Most refugees and migrants arriving in the region wish to move onwards and are commonly subject to returns in violation of due process standards, face inadequate reception conditions, delays in asylum procedures and a lack of long-term solutions prospects. Many of them have experienced and witnessed violence, lost loved ones, faced deprivation and have been separated from their families. An empathic and mentalizing attitude, secure sheltering, addressing health, and educational needs can create a sense of stability and confidence.

Activities of all professionals and organizations working with children in these circumstances must apply the Principles of Best Interests identified in the UN Convention on the Rights of the Child (CRC; Article 3). All those working with children, adolescents, and their families, should safeguard their rights to be heard and to participate in decisions that concern them. An acceleration of such processes will help the youth adjust in a better way.

Children and youth reaching destination countries should be supported in integration into society and provided with mainstream services, as well as offered opportunity to enroll in regular education that is non-discriminatory and appropriately culturally sensitive. At the same time, refugee children and youth should be subject to professional assessment and provided with any necessary additional support. A successful educational, cultural, and religious (if applicable) integration is the key cornerstone for future mental health and the prevention of behavioral disorders. To meet current challenges, there is a need to adopt a public health approach, entailing more frequent use of screening, stepped care, task sharing, task shifting in the current structures, health care financing, and ways of working. Enhancing cultural competence of professionals and monitoring refugees' access and utilization of services is also needed. Special emphasis should be given to understanding refugees' experiences and challenges within the new environment and toward fostering resilience among individuals and communities.

## Data Availability Statement

The original contributions presented in the study are included in the article/supplementary material, further inquiries can be directed to the corresponding author/s.

## Ethics Statement

The studies involving human participants were reviewed and approved by Ethics Committee of Institute of Mental Health, Belgrade. Written informed consent to participate in this study was provided by the participants' legal guardian/next of kin.

## Author Contributions

JV and RG contributed with the research, interpretation and writing of the paper, reviewing, and technical work. OT contributed with technical support and statistical analysis. MP contributed with literature research, analysis, expertise, interpretation, writing, and reviewing process. All authors contributed to the article and approved the submitted version.

## Funding

The authors received funding for this research from the organization UNICEF Serbia.

## Conflict of Interest

The authors declare that the research was conducted in the absence of any commercial or financial relationships that could be construed as a potential conflict of interest.

## Publisher's Note

All claims expressed in this article are solely those of the authors and do not necessarily represent those of their affiliated organizations, or those of the publisher, the editors and the reviewers. Any product that may be evaluated in this article, or claim that may be made by its manufacturer, is not guaranteed or endorsed by the publisher.
